# Establishing BioE3 centres: catalyzing India’s biomanufacturing transformation

**DOI:** 10.3389/fbioe.2025.1681476

**Published:** 2025-09-10

**Authors:** Dhiraj Kumar, Jitendra Kumar

**Affiliations:** Biotechnology Industry Research Assistance Council (BIRAC), New Delhi, India

**Keywords:** BioE3 policy, biomanufacturing, biotechnology infrastructure, translational innovation, bioeconomy, BIRAC, policy implementation, India

## Abstract

India’s bioeconomy is poised for rapid expansion, targeting a valuation of $300 billion by 2030. The recently approved BioE3 Policy (Biotechnology for Economy, Environment, and Employment) aims to reposition India as a global leader in sustainable, high-performance biomanufacturing. In this context. a national stakeholder meeting was convened with ∼75 participants, including CEOs, COOs, and senior representatives from bioincubators, Centres of Excellence, startups, and scientific institutions. Structured group discussions and expert inputs focused on operationalizing BioE3 Centres—new-generation incubation and biomanufacturing hubs aimed at bridging critical infrastructure and capability gaps]. Discussions identified the need for pilot-scale infrastructure, regulatory-grade facilities, skilled human resources, and integration with existing policy mechanisms such as the Biotechnology Industry Partnership Programme (BIPP) and the Entrepreneur-in-Residence (EIR) initiative under BIRAC. Once fully operational, BioE3 Centres are projected to support over 250 startups and MSMEs, generate an estimated 10,000 skilled jobs, and contribute up to $25 billion to India’s targeted $300 billion bioeconomy by 2030. This article outlines the key insights, recommendations, and phased implementation roadmap from the consultation, with implications for national policy execution and global positioning of India’s bioeconomy.

## 1 Introduction

India’s biotechnology sector is experiencing an inflection point, driven by pressing global challenges and national development priorities (Department of Biotechnology, 2024; BIRAC Annual Report, 2023; India’s first Biomanufacturing Institute, 2024; Lalitha, 2025). In August 2024, the Government of India approved the BioE3 Policy, envisioning biomanufacturing as a strategic lever for economy, environment, and employment (Department of Biotechnology, 2024). The policy aims to transform resource-intensive, extractive manufacturing paradigm into a regenerative, circular, and bio-based production model. Key enablers under this framework include the Biofoundry network, National Biomanufacturing Institute (Mohali), and the upcoming BioE3 Centres.

While over 100 incubation centers have been established under BIRAC’s BioNEST scheme, most remain focused on early-stage proof-of-concept development. The transition from lab-scale innovation to commercial-ready manufacturing remains constrained by the absence of pilot infrastructure, regulatory compliance pathways, and integrated mentorship (BIRAC Annual Report, 2023). The concept of BioE3 Centres is designed to address these bottlenecks by enabling translational pathways, pilot-scale validation, and regulatory-compliant biomanufacturing support (India’s first Biomanufacturing Institute, 2024).

## 2 Purpose of the stakeholder meeting

Held on 12 June 2025 in New Delhi the meeting brought together ∼75 stakeholders, including CEOs, COOs, and senior leadership from bioincubators and Centres of Excellence across India, along with representatives from startups and scientific institutions. Through structured group discussions and expert inputs, participants worked to:

- Define the functional scope of BioE3 Centres;
- Identify infrastructure and technical needs for scalable biomanufacturing;
- Explore sustainable business and funding models; with emphasis on PPP co-funding mechanisms
- Deliberate on governance frameworks, including linkages with State-level BioE3 Cells; to clarify whether their role should be advisory, operational, or financial
- Align BioE3 Centres with national missions such as Net Zero and Mission LiFE highlighting the need for biomanufacturing to contribute to sustainability goals.

## 3 Key discussion outcomes

### 3.1 Infrastructure gaps and recommendations

Participants highlighted the limited availability of pilot-scale facilities, regulatory-grade labs, and technology validation platforms in the existing ecosystem (BIRAC Annual Report, 2023).

Gap areas specifically noted included:

• Insufficient availability of National Accreditation Board for Testing and Calibration Laboratories (NABL)/Good Laboratory Practice (GLP)/Good Manufacturing Practice (GMP)-certified laboratories.
• Lack of integrated cleanrooms, fermentation suites, and downstream processing units to support TRL three to eight.
• Inadequate facilities for preclinical safety, toxicity studies, and bridging the “valley of death” between TRL three to eight and commercial deployment.
• Fragmented infrastructure, with duplication in some regions and critical absence in others, especially in the Northeast and eastern India

Recommended features of BioE3 Centres include:

- NABL/GLP/GMP certified laboratories;
- Cleanrooms, fermentation suites, and downstream processing units;
- Facilities for toxicity studies and TRL three to eight support.

To address geographic inequity, a cluster-based deployment strategy was emphasized, aligning centres with regional industrial strengths while ensuring balanced distribution across underserved regions (Lalitha, 2025).

### 3.2 Financial and operational sustainability

To ensure the long-term financial and operational sustainability of BioE3 Centres, there was strong consensus on the need for sustained capital (CAPEX; capital expenditure) and operational (OPEX; operational expenditure) expenditure support, particularly during the initial years of operation. Embedding public–private partnership (PPP) co-funding from the outset—rather than introducing it later—was identified as essential to securing industry commitment and ensuring shared ownership and responsibility. A recommended approach involves government anchor funding to support CAPEX, particularly for establishing regulatory-grade infrastructure, while OPEX can be co-funded by industry through mechanisms such as cost-sharing, user fees, and service contracts.

Diversified revenue models will also be key to sustainability. These may include membership fees, training programs, and fee-for-service offerings such as fermentation, regulatory testing, and other value-added services. Learning from successful PPP and financial models both in India and internationally can guide the BioE3 framework. For instance, India’s BIRAC BIPP program demonstrates a 50:50 government-industry cost-sharing approach that balances public investment with commercial viability. The UK’s Catapult Centres follow a “three-stream” model—combining core government grants, collaborative industry research, and fee-based services—to maintain financial health. Since their inception, Catapult Centres have supported over 6,000 companies, accelerated more than 200 collaborative R&D projects, and leveraged over £2 billion in co-investment, demonstrating the effectiveness of this diversified funding model (House of Lords Science and Technology Committee, 2021). Similarly, the U.S.-based Manufacturing USA Institutes, like NIIMBL (National Institute for Innovation in Manufacturing Biopharmaceuticals, USA), operate under cooperative agreements where industry must at least match federal funding and participate in tiered membership programs with scalable benefits Since its establishment in 2017, NIIMBL has mobilized over $600 million in combined federal and industry investment, supported more than 100 collaborative projects, and developed nationally recognized workforce training programs, underscoring the effectiveness of this co-investment model (National Institute for Innovation in Manufacturing Biopharmaceuticals NIIMBL, 2023). BioE3 Centres can adapt these models by establishing co-funding requirements, engaging industry partners early, and offering flexible membership and service-based revenue streams to ensure operational and financial resilience.

### 3.3 Skill development and entrepreneurial support

Recommendations included: Participants identified workforce shortages in regulatory sciences, IP management, technology transfer, and commercialization. Hiring domain specialists is essential, but equally important is building structured capacity-building programs. Suggested measures included:

• Recruitment of regulatory experts, IP professionals, and commercialization managers.
• Launch of Entrepreneur-in-Residence (EIR) programs to nurture biotech founders with hands-on incubation and scale-up support.
• Development of workforce training modules modeled on international best practices, such as the NIIMBL program, which emphasizes GMP compliance, workforce credentialing, and industry-relevant skills (National Institute for Innovation in Manufacturing Biopharmaceuticals NIIMBL, 2023). NIIMBL has successfully trained over 5,000 professionals in biopharmaceutical.
• Partnerships with universities and Centres of Excellence to create pipelines of skilled graduates aligned with BioE3 thematic clusters (BIRAC Annual Report, 2023; Lalitha, 2025).

### 3.4 Policy and programmatic integration

Stakeholders emphasized integration with multiple national programs to ensure smooth translation from lab to market. Specifically, participants noted the importance of:

• BIPP (Biotechnology Industry Partnership Programme): A flagship PPP initiative that supports high-risk, industry-led R&D through cost-sharing.
• SBIRI (Small Business Innovation Research Initiative): A program supporting early-stage, high-risk research by SMEs in biotechnology. SBIRI has enabled dozens of SMEs to transition from risky early research to viable biotech ventures, catalyzing commercialization pathways and de-risking innovation for small companies (BIRAC Annual Report, 2023).
• ETA (Early Translation Accelerator) Program: An initiative focusses on catalyzing transformation of young academic discoveries with possible commercial and societal impact into economically viable ventures and technologies. Several ETA-supported projects have already progressed to proof-of-concept validation and industry partnerships, demonstrating its effectiveness in bridging the lab-to-market gap (BIRAC Annual Report, 2023).
• State-level BioE3 Cells: Regional arms envisioned to provide advisory, operational, and financial roles in aligning BioE3 Centres with local industrial and academic strengths.

Participants stressed that without such integration, BioE3 Centres risk duplicating efforts or operating in silos. Linking with these national and state-level programs will enable coordinated governance, smoother funding pathways, and alignment with India’s overarching missions such as Net Zero and Mission LiFE.

## 4 Proposed BioE3 centre framework

### 4.1 Thematic areas and regional clusters

Centres should focus on thematic domains based on regional strengths, Rather than duplicating infrastructure across locations, BioE3 Centres should leverage existing industrial and academic strengths within regions. This cluster-based strategy ensures balanced national coverage, avoids fragmentation, and enhances cost-effectiveness (Lalitha, 2025), (Table 1: Thematic focus area for BioE3 Centers).

**TABLE 1:** Proposed thematic focus areas for BioE3 Centres aligned with regional industrial and academic strengths.

Regional hub/State (Illustrative)	Proposed thematic focus Area(s)	Rationale/Existing strengths
Northeast Region (e.g., Guwahati, Imphal)	Agri-biotechnology, Natural Products, Ethnobotany	Rich biodiversity, traditional knowledge base; first BioE3 Cell established in the region
Bengaluru (Karnataka)	Synthetic Biology, Bioinformatics	Strong IT–biotech integration, presence of leading research institutes (NCBS, BBC, IISc, C-CAMP, IBAB)
Hyderabad (Telangana)	Biopharmaceuticals, Vaccine Manufacturing	Established pharma cluster, Genome Valley ecosystem
Pune–Mumbai (Maharashtra)	Industrial Fermentation, Enzymes	Chemical and fermentation industry base, strong academic partners (NCL, ICT, IISER)
Mohali–Chandigarh (Punjab)	Biofoundry, Precision Biomanufacturing	National Biomanufacturing Institute, agricultural biotechnology
Ahmedabad (Gujarat)	Industrial Biotechnology, Bioenergy	Biochemicals and industrial biotech industry clusters
Chennai (Tamil Nadu)	Marine Biotechnology, Bioprocess Engineering	Coastal resources, strong universities (IIT-M, Anna University)
Bhubaneswar (Odisha)	Marine Resources, Aquaculture Biotechnology	Presence of CIBA (Central Institute of Brackishwater Aquaculture), KIIT, and coastal biodiversity
Lucknow (Uttar Pradesh)	Agricultural Biotechnology, Bioinputs	ICAR institutes, seed and agri-bio startups
Delhi NCR	Translational Research, Regulatory Sciences.	Policy hubs, DBT/BIRAC headquarters, translational centres

This clustering model will also enable region-specific specialization ensuring complementarity rather than competition among Centres.

### 4.2 Governance and partnerships

BioE3 Centres are envisioned to be hosted at BioNEST incubators or research institutes, governed through a nodal structure under BIRAC with strategic partnerships with academic institutions, industry, and State BioE3 Cells (BIRAC Annual Report, 2023; India’s first Biomanufacturing Institute, 2024). To ensure transparency, accountability, and financial sustainability, governance will involve multiple layers:

• National Level (BIRAC): Provide strategic oversight, policy alignment, and anchor funding support.
• State-level BioE3 Cells: Play complementary roles:
o *Advisory:* Guide thematic priorities based on regional strengths.
o *Operational:* Facilitate linkages with state innovation agencies, universities, and incubators.
• Industry Partners: Essential from beginning through PPP co-funding (CAPEX and OPEX sharing), joint projects, and membership models.

**FIGURE 1:**
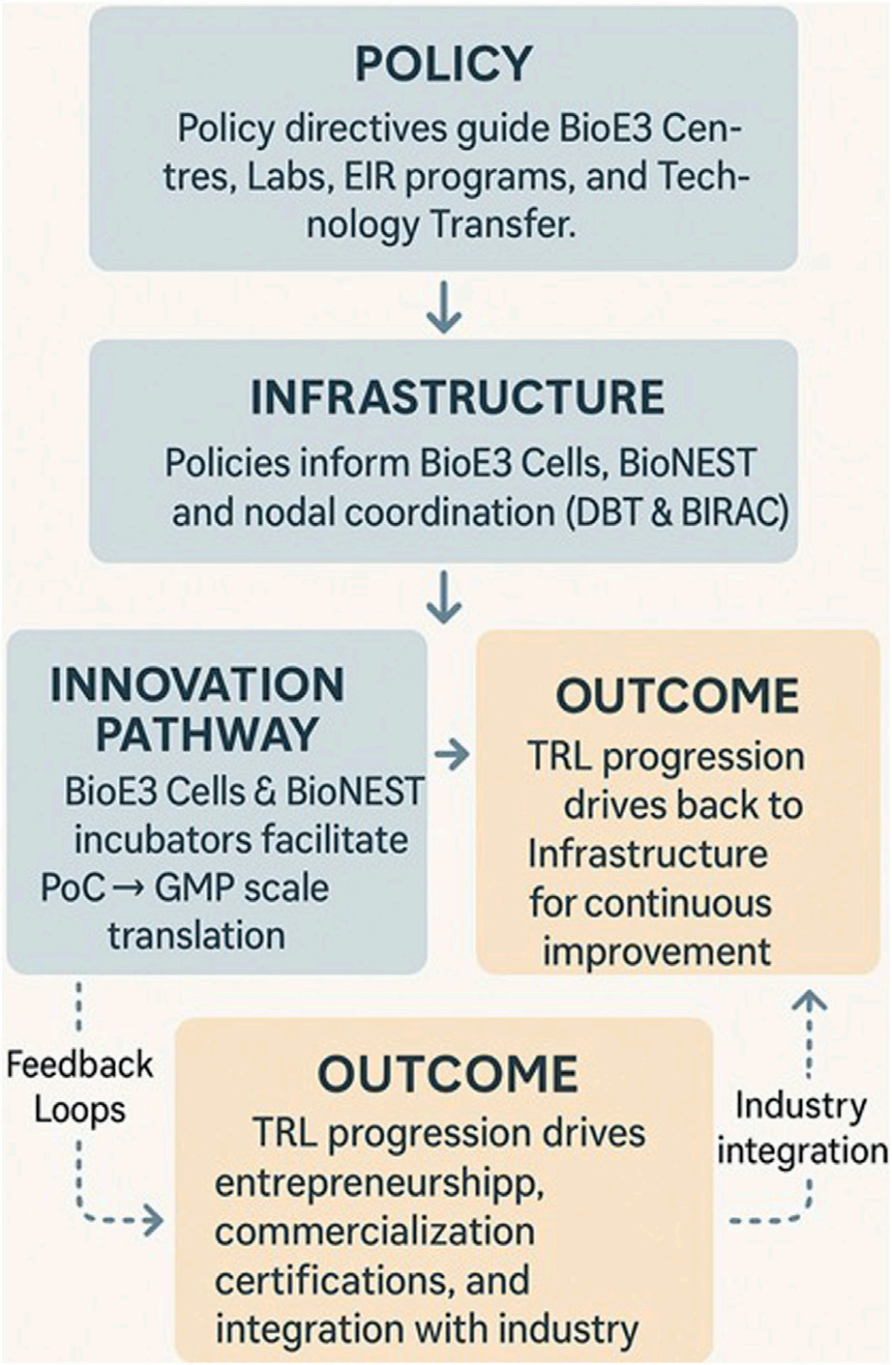
Proposed schematic of the BioE3 ecosystem. Note: The schematic illustrates the BioE3 ecosystem, highlighting the interplay between BioE3 Centres, regional BioE3 Cells, industry partners, and national programs. It depicts how BioE3 Centres serve as hubs for translational research, pilot-scale validation, and regulatory support, while State-level Cells provide regional linkages. Public–private partnerships (PPP) ensure financial sustainability, and integration with national missions (Net Zero, Mission LiFE) positions biomanufacturing as a driver of India’s bioeconomy growth.

PPP models should combine government anchor grants with industry cost-sharing and service revenues. Similar blended models in India’s BIPP program and the UK’s Catapult Centres have already demonstrated sustainability, attracting over 100 PPP projects and £2 billion in leveraged co-investment (BIRAC Annual Report, 2023; House of Lords Science and Technology Committee, 2021). (Figure 1: proposed schematic of the BioE3 ecosystem).

## 5 Recommendations and roadmap

### 5.1 Recommendations include

- Establish cluster-based BioE3 Centres;
- Build scale-up facilities with certification (GLP, GMP, NABL);
- Provide blended CAPEX (government anchor funding) and OPEX (shared through PPP co-funding, user fees, and service contracts) for 3–5 years;
- Launch EIR, IP, and regulatory support programs;
- Link with BioE3 Cells and national missions;
- Embed commercialization and market readiness support (Department of Biotechnology, 2024; BIRAC Annual Report, 2023: India’s first Biomanufacturing Institute, 2024; Lalitha, 2025).

### 5.2 Implementation Timeline

Over the next 5 years, a step-by-step plan will be followed to build a strong BioE3 network in India. In the beginning, five Centres will be set up in key areas with full funding for setup and shared support from government and private partners for running costs. Expert teams will be hired to handle technology, regulations, and business development. Programs will also be launched to support startups, manage patents, and help with approvals. Based on what works well, the network will grow to 8–10 Centres, with better systems for partnerships and some cost-sharing through services and memberships. These Centres will also work closely with State governments and national missions like Net Zero and Mission LiFE. By the end of 5 years, the goal is to have a strong, well-connected network that supports innovation and helps make India a global leader in green and sustainable biomanufacturing.

To ensure robustness of the BioE3 framework, additional consultations are planned, including dedicated meetings with BIRAC and DBT officials. These engagements will focus on validating the stakeholder recommendations, refining the implementation roadmap, and guiding the phased rollout of BioE3 Centres across India.

## 6 Conclusion

The establishment of BioE3 Centres is a pivotal step in reshaping India’s biotechnology innovation architecture. By providing certified pilot-scale facilities, workforce training aligned with GMP standards, and co-funding mechanisms with industry, these Centers will directly support startups and SMEs in advancing 50–100 biomanufacturing innovations to regulatory readiness by 2030. Their success will depend on robust governance, meaningful PPP co-funding, thematic clustering, and strong interlinkages with State-led BioE3 Cells. Together, these features will shape a globally competitive Indian bioeconomy and help achieve the $300 billion bioeconomy target by 2030.
